# Amplicon deep sequencing of *kelch13* in *Plasmodium falciparum* isolates from Senegal

**DOI:** 10.1186/s12936-020-03193-w

**Published:** 2020-03-30

**Authors:** Amy Gaye, Mouhamad Sy, Tolla Ndiaye, Katherine J. Siddle, Daniel J. Park, Awa B. Deme, Aminata Mbaye, Baba Dieye, Yaye Die Ndiaye, Daniel E. Neafsey, Angela Early, Timothy Farrell, Mamadou Samb Yade, Mamadou Alpha Diallo, Khadim Diongue, Amy Bei, Ibrahima Mbaye Ndiaye, Sarah K. Volkman, Aida Sadikh Badiane, Daouda Ndiaye

**Affiliations:** 1 Laboratory of Parasitology and Mycology, Aristide le Dantec Hospital, Cheikh Anta Diop University, Dakar, Senegal; 2 The Broad Institute of MIT and Harvard, Cambridge, MA USA; 3 Department of Immunology and Infectious Diseases, Harvard T. H. Chan School of Public Health, Boston, MA USA; 4 Yale School of Public Health, Laboratory of Epidemiology and Public Health, 60 College Street, New Haven, CT 06510 USA

**Keywords:** PfK13-propeller, Amplicon deep sequencing, Artemisinin resistance, Senegal

## Abstract

**Background:**

In 2006, the Senegalese National Malaria Control Programme recommended artemisinin-based combination therapy (ACT) with artemether–lumefantrine as the first-line treatment for uncomplicated *Plasmodium falciparum* malaria. To date, multiple mutations associated with artemisinin delayed parasite clearance have been described in Southeast Asia in the *Pfk13* gene, such as Y493H, R539T, I543T and C580Y. Even though ACT remains clinically and parasitologically efficacious in Senegal, the spread of resistance is possible as shown by the earlier emergence of resistance to chloroquine in Southeast Asia that subsequently spread to Africa. Therefore, surveillance of artemisinin resistance in malaria endemic regions is crucial and requires the implementation of sensitive tools, such as next-generation sequencing (NGS) which can detect novel mutations at low frequency.

**Methods:**

Here, an amplicon sequencing approach was used to identify mutations in the *Pfk13* gene in eighty-one *P. falciparum* isolates collected from three different regions of Senegal.

**Results:**

In total, 10 SNPs around the propeller domain were identified; one synonymous SNP and nine non-synonymous SNPs, and two insertions. Three of these SNPs (T478T, A578S and V637I) were located in the propeller domain. A578S, is the most frequent mutation observed in Africa, but has not previously been reported in Senegal. A previous study has suggested that A578S could disrupt the function of the *Pfk13* propeller region.

**Conclusion:**

As the genetic basis of possible artemisinin resistance may be distinct in Africa and Southeast Asia, further studies are necessary to assess the new SNPs reported in this study.

## Background

In 2006, the Senegalese National Malaria Control Programme (NMCP) recommended artemisinin-based combination therapy (ACT) with artemether–lumefantrine as the first-line treatment for uncomplicated *Plasmodium falciparum* malaria [1]. The recent emergence of *P. falciparum* resistance to artemisinin derivatives in Southeast Asia has challenged malaria control and elimination efforts in this region [2]. Artemisinin resistance was first reported in western Cambodia in 2008–2009 [3]. This partial resistance, affecting only ring-stage parasites, leads to delayed parasite clearance, though the majority of patients are still able to clear their infections following treatment with an effective partner drug or with an artesunate treatment lasting 7 days [2]. Although ACT remains clinically and parasitologically efficacious in Senegal, the spread of resistance is possible as shown by the spread of resistance to chloroquine and sulfadoxine–pyrimethamine (SP) from Southeast Asia to Africa [3]. This worrying possibility makes ongoing surveillance critical for early detection of any emerging resistance.

The artemisinin-resistant phenotype reported in Southeast Asia and, more recently, in South America has been associated with mutations in the propeller domain of the Kelch 13 protein (*Pfk13*) encoded by the gene *PF3D7_1343700* on *P. falciparum* chromosome 13 [4, 5]. These polymorphisms are broadly associated with a survival rate of more than 1% in ring-stage survival assays (RSA) [6]. More specifically, certain non-synonymous mutations in *Pfk13* result in reduced sensitivity of *P. falciparum* to artemisinin, as demonstrated by multiple lines of evidence including laboratory studies of artificially acquired resistance, genetic association studies of natural resistance and allelic replacement experiments [3]. These data support the use of *Pfk13* mutant genotypes as a marker for reduced parasite susceptibility to artemisinin [7]. Due to the implications of these mutations, surveillance for *Pfk13* SNPs have been conducted in many different countries to detect the presence of resistant parasites. To date, multiple resistance-associated mutations in *Pfk13* have been described in Southeast Asia [8]. Surveillance of emerging drug resistance in regions endemic for malaria requires an integrated approach comprising various techniques such as real-time PCR, high-resolution melting (HRM), single-nucleotide polymorphism (SNP)-based custom genotyping assay, pyrosequencing, and Sanger sequencing of a genomic region containing one or more resistance mutations [9].

However, the diversity of mutations involved, and the fact that novel mutations can arise independently in different locations of the gene, or indeed in other genes, make it difficult to track the emergence of resistance using conventional molecular marker approaches [10]. Additionally, all these conventional methods are not able to detect polymorphisms at low minor allele frequencies (MAFs) [11], which requires the implementation of a much more sensitive tool, such as next-generation sequencing (NGS) that can detect mutations with a MAF as low as 1%. This sensitivity is particularly important in the context where treatment with an anti-malarial drug can exert a selective pressure on minor populations of drug resistant parasites [12]. Recent advances in NGS methods have further made these technologies cheaper and more feasible to implement in malaria endemic areas, outside of traditional large genotyping centers, where surveillance is most needed.

Developing molecular surveillance tools is crucial for early detection of circulating reportable and non-reportable drug resistance alleles in low frequency. The early detection of those alleles before they become fixed and spread throughout the parasite population is a public health concern. Deep sequencing of malaria parasites is an efficient approach for quantifying drug-resistance alleles and is more adaptable for large-scale drug-resistance surveillance [7, 13], thus capacity should be established to perform this locally in malaria endemic countries to enable surveillance in real-time.

Here an amplicon-based sequencing approach was implemented locally in Senegal for malaria drug resistance surveillance. This approach was used for *Pfk13* drug resistance screening of 81 samples from Senegal, providing updated surveillance data on mutation frequencies from this important gene. This example further serves as a model for future surveillance systems using deep sequencing of targeted genomic regions in malaria-endemic areas to monitor known and novel polymorphisms in *P. falciparum* genes.

## Methods

### Study site and sample collection

The study protocol was approved by the National Ethics Committee for Health Research of Senegal. Before participant recruitment and sample collections were initiated, written and informed consent was obtained from all participants. Following this, venous blood samples were collected in 5 ml vacutainer tubes and filter paper was made for molecular testing. All individuals in this study presented with uncomplicated malaria and parasite presence was confirmed by microscopy.

**Fig. 1:**
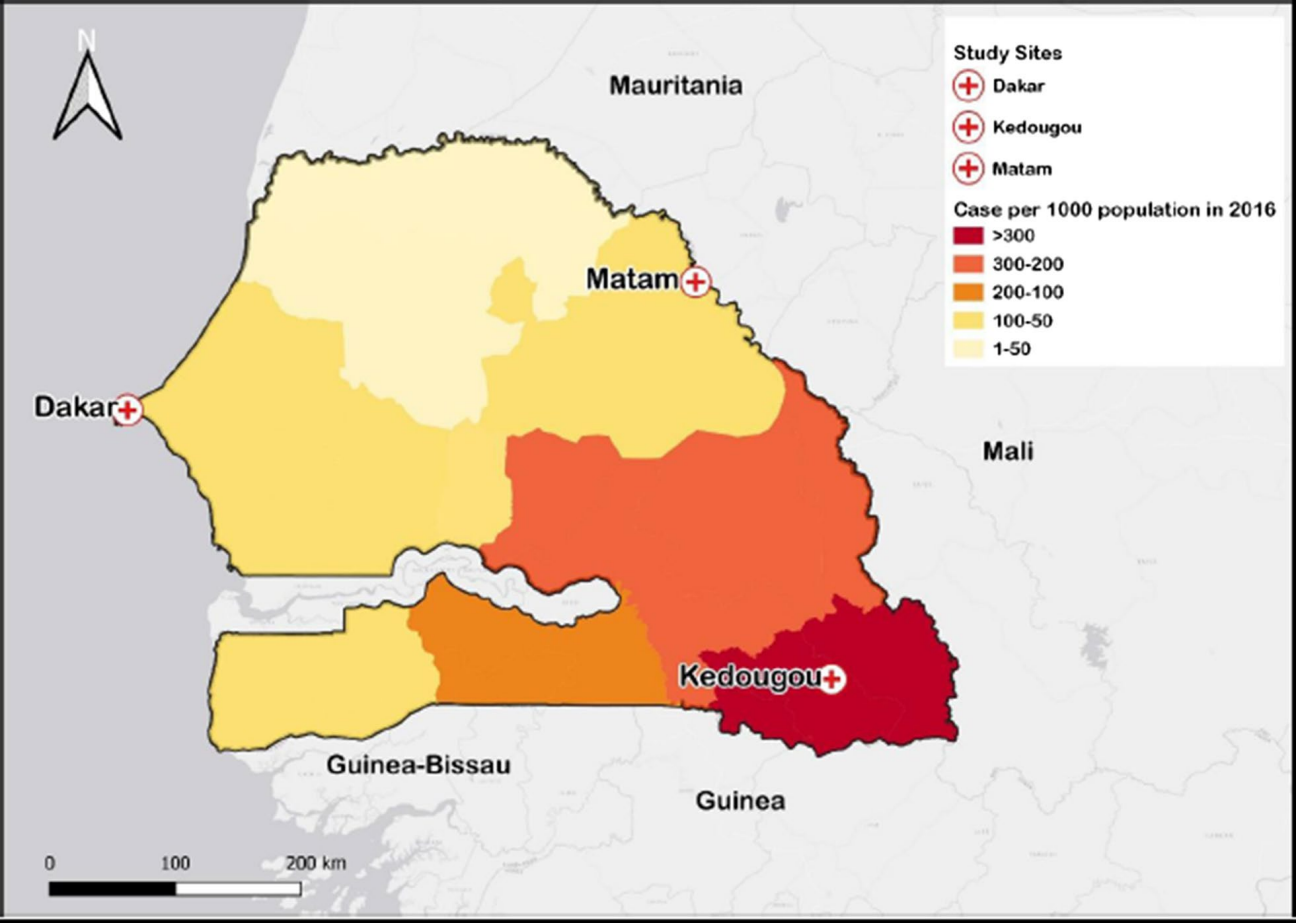
Map of Senegal showing the study sites and the malaria incidence in 2016. Geographic information system (QGIS 3.4.5)

In total, 81 patient samples from three regions of Senegal were selected: Dakar (48), Kédougou (27) and Matam (6), which present different levels of malaria prevalence. In Dakar malaria transmission is low and parasite prevalence is estimated at 1.3% [13]. In the southeastern Kédougou region, malaria is hyper-endemic with an incidence higher than fifteen malaria cases per 1000 habitants [1]. Matam, located in northern Senegal, is a hypo-endemic pre-elimination malaria zone. In Kédougou and Matam, the samples were collected in September 2016 and in Dakar samples were collected between October and November 2015 (Fig. 1).

### Laboratory procedures

#### Gene amplification

DNA was extracted from the filter papers using the QIAamp DNA Blood Mini kit (Qiagen^®^) according to the manufacturer’s instructions. The *Pfk13* gene was amplified by PCR. As a positive control, the NF54 (Wild Type) and the Clone 7 (Mutant) laboratory strains were used, and PCR-grade water was used as a negative template control. Amplification reactions were carried out in 20 μl volumes containing 4 μl Phusion high-fidelity PCR master mix, 0.2 μl HF Phusion Taq Polymerase, 10 mM dNTPs, 10 μM of each forward and reverse primer and 2 μl of template DNA. Primer sequences were previously designed and published by Talundzic et al. [7]: K13Pf_F1:5′GCAAATAGTATCTCGAAT3′,K13Pf_R1:5′CTGGGAACTAATAAAGA3′.

The cycling parameters used to amplify this gene were as follows: 94 °C for 5 min, 35 cycles of (94 °C for 30 s, 46 °C for 1 min and 72 °C for 90 s), with a final extension at 72 °C for 5 min [14]. Amplicons were visualized on a 2% agarose gel stained with ethidium bromide, using a 1-kb Plus DNA ladder GeneRuler (Thermo Fisher Scientific^®^). The amplicon size was 2120 bp. The PCR product from each sample was purified using a 0.6X DNA SPRI (Agencourt AMPure XP beads Beckman Coulter^®^, CA, USA). After purification, the sample was quantified by The Qubit^®^ 3.0 Fluorometer.

#### Library preparation and amplicon sequencing

A total of 1 ng of purified PCR product from each sample was used to prepare sequencing libraries using the Nextera XT Library Preparation kit (Illumina) [15]. DNA libraries were cleaned using a 0.6X DNA SPRI. Sequencing libraries were then quantified by qPCR using the KAPA Library Quantification Kits (Roche) on a Roche LightCycler 96 instrument (Roche Molecular Systems, Inc) according to manufacturer’s instructions [16]. After the library quantification, an equimolar pool of libraries was created and quantified using the Agilent High Sensitivity DNA Kit on the BioAnalyzer (Agilent). Sequencing was performed in 3 batches using the Illumina MiSeq reagent kit v2 platform using 101 bp paired end sequencing. Sample preparation, sequencing and data analysis were performed at the Laboratory of Parasitology and Mycology at Le Dantec Hospital, Dakar, UCAD.

### Data analysis

**Fig. 2:**
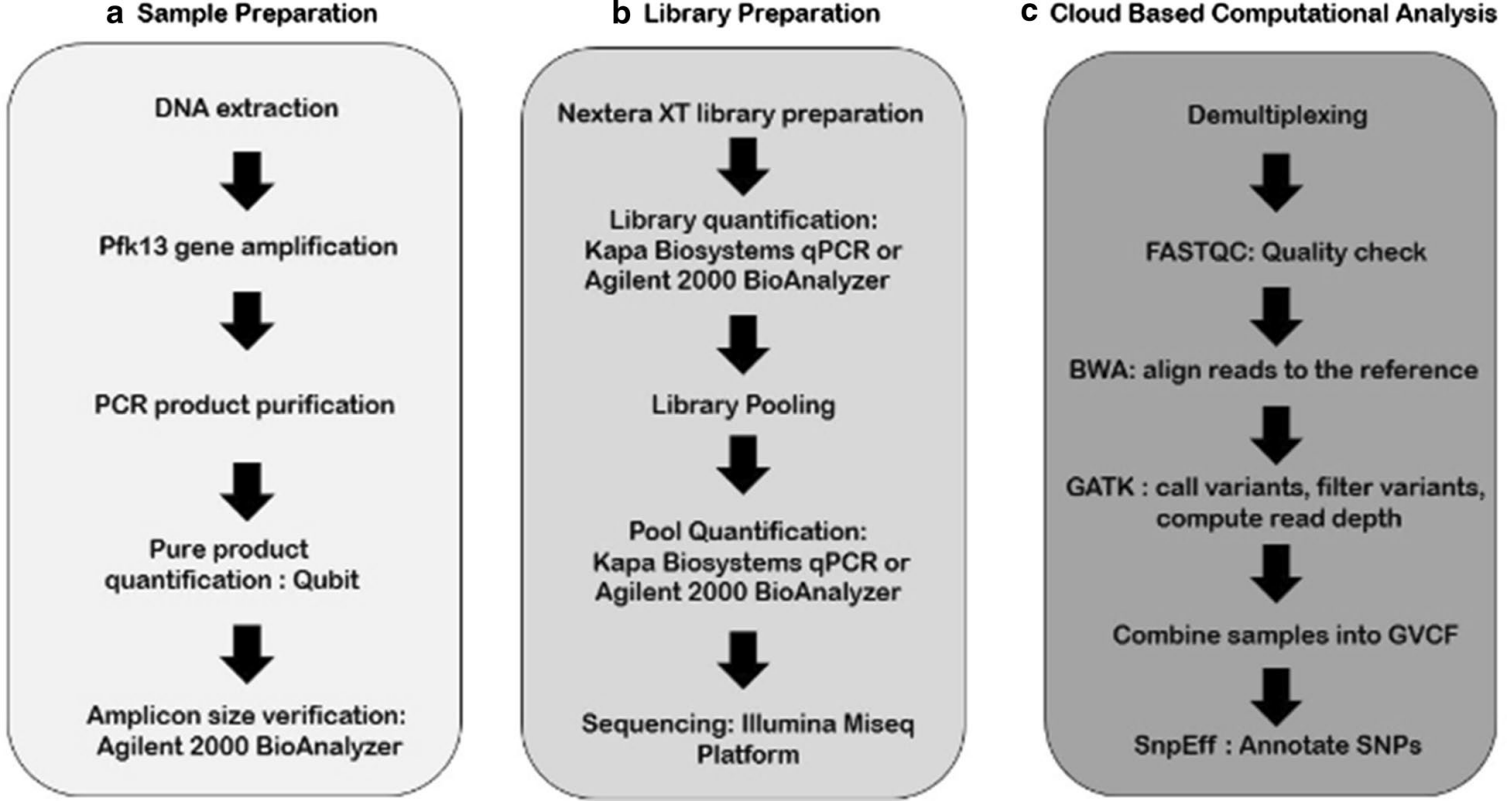
Workflow of data generation and analysis

Sequencing data were analysed using open source software implemented on the DNAnexus cloud platform (https://github.com/dpark01/broad_malaria_firecloud). A computational pipeline for *P. falciparum* SNP (Pf-snp) calling based on the methodology described in the Pf3k project [17] and GATK best practices (https://software.broadinstitute.org/gatk/) was constructed (Fig. 2). Briefly, after demultiplexing and filtering, reads were aligned to the reference genome PlasmoDB38_Pfalciparum3D7_Genome.fasta (https://plasmodb.org/) with BWA-mem, Picard (http://broadinstitute.github.io/picard/) was used for read sorting and marking duplicates and base recalibration was performed with GATK3.6 (software.broadinstitute.org). After processing, variants were detected using HaplotypeCaller in GATK3 and the SNPs were filtered using the following criteria: QualityDepth (QD < 2.0), FS > 60.0, MappingQuality (MQ < 40.0), MQRankSum < − 12.5, ReadPosRankSum < − 8.0, Allele frequency > 2%. These same parameters were also used to identify indels with the following modifications: QD < 2.0, FS > 200.0, ReadPosRankSum < -20.0. PlasmoDB-38_Pfalciparum3D7.gff (https://plasmodb.org/) was used as the annotated reference and SnpEff was used for variant annotation and prediction [18]. The Integrative Genomics Viewer (IGV 2.4.13) [19] was used to visualize all SNP calls and confirm the presence of each reported SNP relative to the reference sequence.

All the SNP data were submitted to the ENA database, with the following accession numbers: PRJEB35317 and ERZ1128412 (https://www.ebi.ac.uk/ena). Variants detected in this study were compared to previously published data from other sub-Saharan African countries including candidate and validated resistance mutations in *Pfk13* from the World Health Organization (WHO) Global Malaria Programme [2].

## Results

**Fig. 3:**
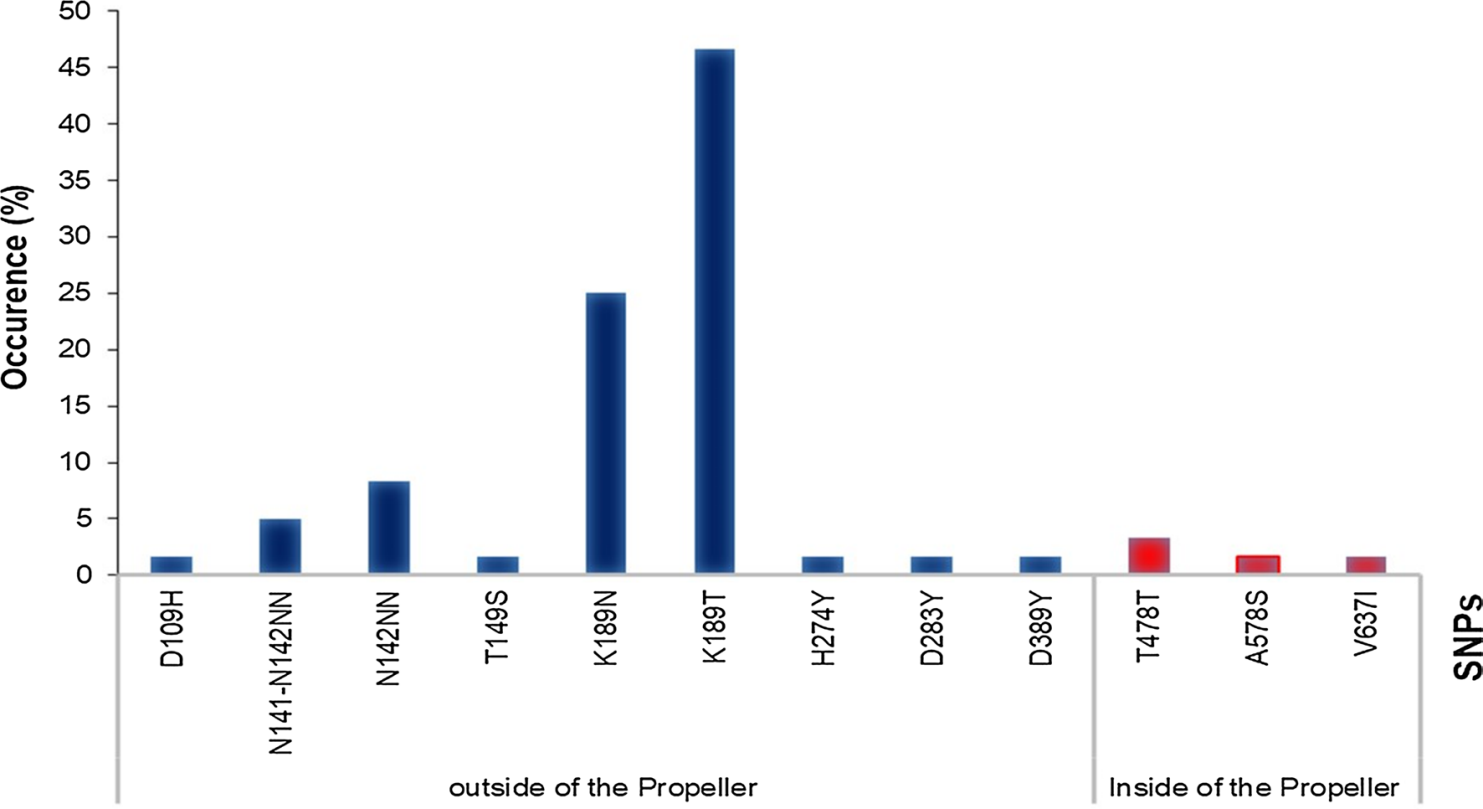
Occurrence of mutations observed in the *Pfk13* gene in *P. falciparum* isolates from Senegal across 81 tested samples. Each bar corresponds to the percentage of samples in which the variant allele was detected

The *Pfk13* gene from 81 samples from three different regions of Senegal with a mean of 341,880 (range 16,348–1,263,000) reads per sample was sequenced. Using the Pf-snp pipeline, 10 SNPs around the propeller domain were identified—nine non-synonymous SNPs and one synonymous SNP—and two insertions. Three of these SNPs were located in the propeller domain at positions 478, 578 and 637 and the remainder were outside of the propeller domain (positions 109, 141,142,149, 189, 189, 274, 283 and 389) (Fig. 3). The most common SNPs identified outside the propeller domain were K189T and K189N, detected in 46.66% and 25% of samples, respectively (Fig. 3). Both of these SNPs were present in all three study sites. The comparison of *Pfk13* polymorphisms identified in this study with those previously identified in sub-Saharan Africa areas showed that 8 of the 12 polymorphisms detected have been previously identified (Table 1). Notably, however, the mutations C580Y, R539T, and Y493H, which have been associated with ART resistance in vitro and/or delayed *P. falciparum* parasite clearance in vivo in Southeast Asia were not detected in any of the samples.

**Table 1:** Mutations in *Pfk13* previously observed in Africa and in the present study

SNPs	2015/2016	2013/2014 [7, 20, 23–26, 29]									2012/2013 [29] [33]	
	This Study	COG (n/N)	ROC (n/N)	GAB (n/N)	GHA (n/N)	IC (n/N)	KEN (n/N)	MAL (n/N)	SEN (n/N)	UGA (n/N)	SEN (n/N)	UGA (n/N)
N141–N142NN	3/81	–	–	–	–	–	–	–	–	–	–	–
N142NN	5/81	–	–	–	–	–	–	–	7/58	–	–	–
T149S	1/81	–	–	–	–	–	–	–	1/58	–	4/64	–
K189N	15/81	–	–	–	–	–	–	–	31/207	1/29	–	–
K189T	28/81	–	–	–	–	–	–	–	130/207	10/29	27/64	–
T478T	2/81	–	–	–	–	1/98	1/108	–	–	–	–	–
A578S	1/81	1/82	2/127	1/93	1/234	–	3/108	1/91	–	1/133	–	–
V637I	1/81	–	–	–	–	–	–	–	–	–	3/92	1/132

“N” is the number of samples sequenced and “n” is the number of samples containing mutant allele

*COG* Congo-Kinshasha, *ROC* Republic of Congo, *GAB* Gabon, *GHA* Ghana, *IC* Côte d’Ivoire, *KEN* Kenya, *MAL* Mali, *SEN* Senegal, *UGA* Uganda

## Discussion

The identification of the *Pfk13* mutations implicated in artemisinin resistance has allowed for real time surveillance of the emergence and spread of this resistance in malaria endemic regions. In order to participate in the surveillance of the potential emergence of artemisinin resistance in Africa, here a deep amplicon sequencing method was used to identify polymorphisms in the *Pfk13* gene in different areas of Senegal. To date, more than 200 non-synonymous mutations in the *Pfk13* gene have been reported [2]. In Southeast Asia and, more recently, South America a number of these mutations have been associated with delayed parasite clearance following artemisinin-based treatments, including C580Y, R539T, Y493H, I543T, F446L, P553L, N458Y, P574L and R561H [2]. In Africa, a number of non-synonymous mutations in *Pfk13* (including T149S, K189N, K189T, A578S, G592V, and V637I) have been identified in malaria-endemic countries from east to west [20]. To date, none of the mutations associated with delayed clearance have been reported in Africa, however, synonymous mutations at the same positions have been reported, such as the P553P mutation reported in Nigeria [21]. In addition, new mutations have been detected, such as the M579I mutation found in Equatorial Guinea that was associated with an increased parasite clearance time on day 3 after dihydroartemisinin-piperaquine (DHA-PIP) treatment [22].

The study of *Pfk13* gene polymorphisms within different areas in Senegal showed an interesting non-synonymous mutation inside the propeller domain (A578S). A578S, found in one sample from the region of Matam with an allele frequency of 2%, has not previously been reported in Senegal, although a different non-synonymous mutation at the same position (A578D) was reported in Thiès [7]. A578S is the most widespread *Pfk13* SNP observed in Africa, having been reported in Mali, Angola, Democratic Republic of the Congo, Uganda, Gabon, Ghana, Congo-Kinshasa, Republic of Congo and Kenya [23–27]. However, it is consistently detected at low frequencies (Table 1). The functional impact of A578S is unclear, but several recent studies have hinted at a potential function. A578S is very close to the C580Y mutation and computational modeling and mutational sensitivity predictions suggest that the A578S SNP could disrupt the function of the propeller domain [27]. Furthermore, in vitro experiments have reported an association with prolonged parasite clearance after treatment with artesunate in Uganda [28]. However, no association of this allele with clinical or in vitro resistance to artemisinin has been shown. In the context of this study, the detection of the A578S mutation in Matam is especially intriguing as Matam is a pre-elimination malaria zone. The anti-malarial immunity is low in low transmission areas making them prone to the emergence of drug resistance.

In the propeller domain the V637I SNP, previously reported in Dakar [29], was also identified. The synonymous mutation T478T previously reported in Côte d’Ivoire was also found [20]. The major mutations identified outside the propeller domain were K189T and K189N. The mutations K189T and K189N were the predominant *PfK13* polymorphisms reported in Senegal and were previously found in Dakar [29] and in Thiès [7]. The NN insert between codons 141 and 142 of *Pfk13* reported here is the first of its kind seen in Senegal (Table 1). However, insertions between codons 142 and 143 have previously been reported in India [30] and insertions at codon 142 of *Pfk13* have been reported in isolates from Senegal [29] and Cambodia [31]. The T149S mutation detected here in Kédougou was also previously reported in Dakar [29]. The role of this mutation is not well established although these mutations seem to be associated with increased parasite clearance, with a half-life > 5 h [32]. For the first time in Africa 4 additional non-synonymous SNPs: D109H, H274Y, D283Y, and D389Y were reported. All 4 SNPs are located outside of the propeller domain. Experimental evaluation of these SNPs in vitro is needed to understand the potential functional implications of these novel, non-propeller-domain mutations.

## Conclusion

The goal of this study was to test and implement amplicon deep sequencing at the laboratory for routine surveillance of malaria cases in Senegal. Target amplicon deep sequencing is an efficient way to quantifying specific polymorphisms of gene in parasite populations in surveillance studies. A cloud-based pipeline to detect SNPs and indels was also implemented, based on established best practices that allows mutations to be identified in less than 10 h after sequencing completion. The presence of the non-synonymous mutation A578S in the *Pfk13*-propeller domain in this study suggest that ongoing surveillance is crucial for early detection of any emerging resistance. Moreover, as artemisinin resistance may emerge independently in Africa it is important to functionally characterize newly reported SNPs, such as those identified in this study. Finally, the capacity to perform and analyse targeted deep sequencing data locally represents an important advance for in-country surveillance and can be readily adapted to other genes and applications.

## Data Availability

Excepted the SNPs data deposit on ENA by this accession numbers: PRJEB35317 and ERZ1128412 (https://www.ebi.ac.uk/ena), all Data used to support the findings of this study are included within the article.

## Abbreviations

NGS: Next generation sequencing
PCR: Polymerase chain reaction
HRM: High resolution melting
SNP: Single nucleotide polymorphism
NMCP: National Malaria Control Programme
WHO: World Health Organization
DNA: Deoxyribonucleic acid
HF: High-fidelity
DNTP: Deoxyribonucleotide triphosphate
SE: Southeast
K13: kelch13
